# QTL and candidate genes for heterophylly in soybean based on two populations of recombinant inbred lines

**DOI:** 10.3389/fpls.2022.961619

**Published:** 2022-08-16

**Authors:** Qiang Chen, Bingqiang Liu, Lijuan Ai, Long Yan, Jing Lin, Xiaolei Shi, Hongtao Zhao, Yu Wei, Yan Feng, Chunji Liu, Chunyan Yang, Mengchen Zhang

**Affiliations:** ^1^Hebei Laboratory of Crop Genetics and Breeding, National Soybean Improvement Center Shijiazhuang Sub-Center, Huang-Huai-Hai Key Laboratory of Biology and Genetic Improvement of Soybean, Ministry of Agriculture and Rural Affairs, Institute of Cereal and Oil Crops, Hebei Academy of Agricultural and Forestry Sciences, Shijiazhuang, Hebei, China; ^2^Hebei Key Laboratory of Molecular and Cellular Biology, Key Laboratory of Molecular and Cellular Biology of Ministry of Education, Hebei Collaboration Innovation Center for Cell Signaling, College of Life Science, Hebei Normal University, Shijiazhuang, China; ^3^CSIRO Agriculture and Food, St Lucia, QLD, Australia

**Keywords:** heterophylly, QTL, soybean, leaf shape, breeding

## Abstract

Heterophylly, the existence of different leaf shapes and sizes on the same plant, has been observed in many flowering plant species. Yet, the genetic characteristics and genetic basis of heterophylly in soybean remain unknown. Here, two populations of recombinant inbred lines (RILs) with distinctly different leaf shapes were used to identify loci controlling heterophylly in two environments. The ratio of apical leaf shape (LSUP) to basal leaf shape (LSDOWN) at the reproductive growth stage (RLS) was used as a parameter for classifying heterophylly. A total of eight QTL were detected for RLS between the two populations and four of them were stably identified in both environments. Among them, *qRLS20* had the largest effect in the JS population, with a maximum LOD value of 46.9 explaining up to 47.2% of phenotypic variance. This locus was located in the same genomic region as the basal leaf shape QTL *qLSDOWN*20 on chromosome 20. The locus *qRLS19* had the largest effect in the JJ population, with a maximum LOD value of 15.2 explaining up to 27.0% of phenotypic variance. This locus was located in the same genomic region as the apical leaf shape QTL *qLSUP*19 on chromosome 19. Four candidate genes for heterophylly were identified based on sequence differences among the three parents of the two mapping populations, RT-qPCR analysis, and gene functional annotation analysis. The QTL and candidate genes detected in this study lay a foundation for further understanding the genetic mechanism of heterophylly and are invaluable in marker-assisted breeding.

## Introduction

Multiple leaf morphologies may exist in a single plant, which is known as heterophylly. It is well known that leaf morphology can be affected by many factors. Due to the temporal development of the shoot apical meristem, changes of leaf morphology may occur during plant development, thus it can be used as a marker of juvenile-to-adult phase transition in some plant species (Telfer et al., 1997; Beydler et al., 2016; Silva et al., 2019). The development of distinct types of juvenile and adult foliage is referred as heteroblasty, and it is one of the most intriguing mechanisms contributing to leaf shape diversification (Chitwood and Sinha, 2016). Different leaf morphologies may also be the result of a rigid development program (Kerstetter and Poethiq, 1998) or a plastic response of plants to different habitat conditions (Titus et al., 2001). In addition, both light intensity and quality modulate leaf shape, size, and thickness (Givnish, 1988; Yano and Terashima, 2001).

Previous studies have shown that the different forms of leaves themselves in a plant may affect light absorption, CO_2_ fixation, and photosynthetic efficiency. For example, it was reported that different leaves of *Sabina vulgaris* have different photosynthetic rates, light compensation points, respiration rates, and water use efficiency (Tanaka-Oda et al., 2010). It was also reported the photosynthetic rate of serrated ovoid leaves was higher than that of ovate and lanceolate leaves and the broad oval leaves have a stronger osmotic capacity than lanceolate leaves in *Populus euphratica* (Liu et al., 2015).

It is also well-known that leaf morphology is a key contributor to canopy structure and affects light distribution, ventilation permeability, and light energy utilization efficiency of a population (Bhagsari and Brown, 1986; Sarlikioti et al., 2011). Heterophylly can allow more efficient use of light energy by affecting canopy structure, gas exchange, and light interception of the lower canopy (Hejnák et al., 2014; Beyschlag and Zotz, 2017). For example, relatively narrow leaves on top of a plant allow more light through to the middle and lower leaves, leading to increased photosynthetic activity throughout the canopy. Relatively bigger and rounder leaves in the middle and lower nodes facilitate absorption of scattered light. Available results strongly suggest that breeding crop varieties for high yield and better quality through optimization of leaf morphology is a feasible strategy (Eshed and Zamir, 1995; Chitwood et al., 2014; Rowland et al., 2020).

Soybean (*Glycine max L.* Merr), one of the most economically important leguminous crops, is a crucial source of plant proteins and oil for both humans and domesticated animals (Graham and Vance, 2003). Due to the increase in market demands, breeding soybeans for higher yields is an urgent priority. Crop yield is an aggregate of complex traits, which is affected by many morphological, physiological, and agronomic traits. Leaves are the major photosynthetic organs in plants, and leaf shape is a major component of plant architecture. It follows, therefore, that suitable leaf shapes may significantly enhance the photosynthetic capacity of soybean plant, and thereby increase yields and potentially improve specific quality traits (Reinhardt and Kuhlemeier, 2002).

Leaf morphologies in soybean can be mainly classified into two categories: ovate and lanceolate (Baldocchi et al., 1985; Jun et al., 2014; Xia et al., 2018; Wang et al., 2019). More than 70 quantitative trait loci (QTL) associated with soybean leaflet shape have been reported.^1^ One single recessive gene *ln* was found to control lanceolate leaf formation, and its dominant allele, *Ln*, encodes ovate leaf formation (Fang et al., 2013). In addition, several genes have been predicted to regulate leaf morphology (Liu et al., 2019; Wang et al., 2019). However, available reports are all focused on studying single types of leaf morphology and none has considered the genetic mechanism of heterophylly. In the study reported here, we investigated the genetics of heterophylly in soybean by analyzing two populations of recombinant inbred lines (RILs). There were three main objectives in this study, and they included: (1) to clarify the genetic characteristics of heterophylly; (2) to identify major QTL controlling heterophylly; and (3) to predict candidate genes responsible for heterophylly in soybean.

## Materials and methods

### Plant materials

**Figure 1:**
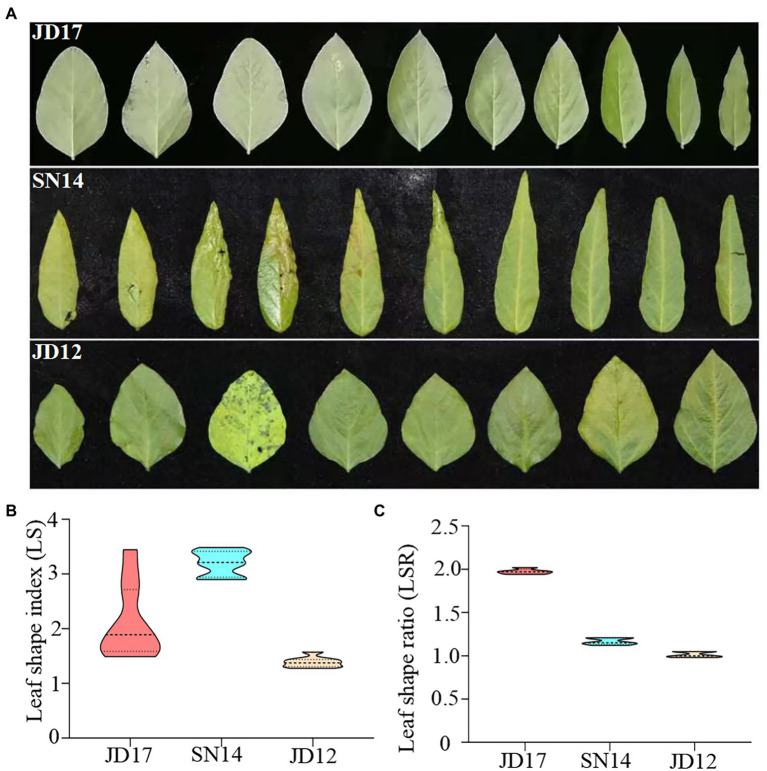
Phenotypic variation in leaf shape among the three parental genotypes at the developmental stage of R6. **(A)** Trifoliate leaves of the first ten leaves (1^st^ trifoliate leaves on the left); **(B)** the distribution of leaf shape index (LS); and **(C)** leaf shape ratio of whole plants.

Two RIL populations (F_6:10_) were generated and used in this study. They were generated from three cultivars with contrasting leaf morphology phenotypes: Jidou17 (JD17, heterophylly), Jidou12 (JD12, ovate leaves), and Suinong14 (SN14, lanceolate leaves). JD17 is a high-yielding cultivar from the Institute of Cereal and Oil Crops at the Hebei Academy of Agricultural and Forestry Sciences. It is adapted to the Huang-Huai-Hai region. This variety displays a remarkable diversity in leaf shape within single plants at the full seed reproductive growth stage (R6), with the leaf shape from bottom to top on the main stem gradually changing from oval to narrow (Figure 1A).

The two RIL populations were constructed with JD17 as a common parent. JD12 from Hebei Province is characterized by ovate fractures of leaflets at R6. SN14 from Heilongjiang Province is characterized by narrow fractures of leaflets at R6. The two RIL populations were designated as JJ (JD17 × JD12) and JS (JD17 × SN14), respectively. Both populations were derived by advancing F2 lines to six generations using the method of single seed descent (SSD). JJ and JS consisted of 191 and 271 RIL lines, respectively.

### Field trials and trait evaluation

The two RIL populations and the three parents were planted in Shijiazhuang of Hebei Province for two consecutive seasons, in 2017 and 2018, respectively. All the RILs in each population were arranged in a randomized complete block design with three replicates. Each plot contained three rows of 2.0 m in length with a row-spacing of 0.5 m. Planting density was 20 seeds per row. To control experimental error, each of the replicates was sown in a square block and three adjacent blocks were used for each of the populations. The block sizes were about 750 and 1,050 square m for the JJ and JS populations, respectively.

At R6, three plants in the middle row were harvested from each plot. Leaf characteristics were measured based on the three most basal leaflets and the top three fully developed leaflets on the main stem from each plant. The leaf length (LL) and leaf width (LW) of the top and basal leaves of each plant were measured. Leaf shape index of the top leaf (LSUP) and leaf shape index of the base leaf (LSDOWN) were obtained separately by calculating the ratio of LL to LW (Dinkins et al., 2002). The ratio of the leaf shape index (RLS), and that of LSUP to LSDOWN was calculated to quantify the heterophylly of each RIL and the parents.

### Statistical analysis

The average values from the nine plants (3 plant/plot × 3 replicates) for each RILs were employed in the subsequent analysis. Statistical analysis was performed using SPSS Statistics 17.0. The phenotypic data of the two populations were analyzed using ANOVA. A mixed procedure was used with genotype and location included as fixed effects in estimating heritability. A *post hoc* Turkey’s test was used to compare means. The broad-sense heritability of single environments was then calculated with the following formula:


$$H^{2}=\sigma^{2}G/\left(\sigma^{2}G+\sigma^{2}GE/n+\sigma^{2}E/nr\right)$$

where *σ*^2^*G* is the genotypic variance; *σ*^2^*e* the error variance, *σ*^2^*GE* is genotype x environment interaction variance, n being the number of environments and r the number of replications (Smith et al., 1998).

### GBS library construction and SNP identification

Genomic DNA was extracted using the NuClean Plant Genomic DNA Kit (CWBIO, Beijing, China) following the manufacturer’s protocol. SNP genotyping was performed using a GBS approach. The GBS library was constructed as previously described (Cheng et al., 2015). Paired-end sequencing was performed on selected tags using the Illumina 2,500 platform (Illumina, United States) at the Novogene Bioinformatics Institute, Beijing, China.

The Burrows-Wheeler Aligner (BWA), SAMtools, and a custom Perl script were used to identify SNPs in the RIL populations (Zhou et al., 2016). The software ANNOVAR (Wang et al., 2010) was used to align and annotate SNPs or InDels based on the GFF3 files of the soybean genome annotations in the Phytozome database.^2^

### Bin map construction and QTL analysis

High-quality SNP markers were identified using the following criteria: (i) average sequence depths should be >10-fold in the parents and (ii) markers with more than 20% missing data were excluded. Parental allele assignment and imputation were performed using the Window LD function of FsFHap (Swarts et al., 2014) incorporated into TASSEL 5.0.

After filtering the GBS data, redundant markers were binned based on segregation patterns deciphered in the RIL populations using the BIN function in IciMapping 4.1 (Li et al., 2008; Meng et al., 2015). Markers segregating with at least one other marker were retained, and one marker was selected to represent each bin (Zeng et al., 2019). The selected markers were used for linkage map construction using the MAP foundation for statistical computing using the IciMapping Version 4.1 software.^3^

Additive QTL was detected using inclusive composite interval mapping (ICIM) in the BIP (bi-parental populations) model of QTL IciMapping software v4.1, with the *p* values for entering variables (PIN) = 0.01. The threshold of the logarithm of the odds (LOD) score for evaluating the statistical significance of QTL effects was determined using 1,000 permutations at the significance level of 0.05 (Pei et al., 2018).

QTL-by-environment interaction analysis was conducted using the MET functional module in QTL IciMapping V4.1 with the following parameters: Step was 5.0 cM, PIN was 0.0001, and Type I error was 0.05 in 1,000 permutation tests (Times). These were used to estimate the interactive effects between additive QTL and environments, as described by Meng et al. (2015).

### Parental resequencing and candidate gene annotation

The genomic DNA of JD17, JD12, and SN14 were extracted from the young leaves of 4-week-old soybean plants. DNA extraction was performed using the cetyltrimethylammonium bromide (CTAB) method. Libraries for each of the varieties were constructed using the TruSeq Library Construction Kit (Illumina Inc., United States) with an insert size of approximately 350 bp. Sequencing was conducted on the Illumina HiSeq platform by Novogene, Beijing, China. The original image data generated by the sequencing machine were converted into sequence data *via* base calling (Illumina pipeline CASAVA v1.8.2) and then subjected to a quality control (QC) procedure to remove unusable reads: (1) Reads contain the Illumina library construction adapters; (2) reads contain more than 10% unknown bases (N bases); and (3) one end of the reads contains more than 50% of low-quality bases (sequencing quality value ≤5). Reads were aligned to the Wm82. a2. v1 (soybean reference /genome) from SoyBase^4^ using BWA with default parameters (Li and Durbin, 2009). Subsequent processing, including duplicate removal, was performed using SAMtools and PICARD^5^ (Li et al., 2009). The raw SNP/InDel sets are called by SAMtools with the parameters as ‘-q 1 -C 50 -m 2 -F 0.002 -d 1000’. We then filtered the data using the following criteria: (1) The mapping quality >20; and (2) The depth of the variate position >4.

### RNA isolation and RT-qPCR

Soybean cultivars JD17, JD12, and SN14 were planted in Shijiazhuang of Hebei Province in 2021, and the terminal buds at vegetative growth stage (V2) and R6 were used for RNA extraction. Total RNAs were isolated using FastPure® Plant Total RNA Isolation Kit (Vazyme, Nanjing, China). Then, 1,000 ng RNA was used for cDNA synthesis with HiScript®IIQRT SuperMix (Vazyme, Nanjing, China). Primers were designed using Primer Premier 5 (Supplementary Table S7), and those for the *Ln* gene were described earlier (Fang et al., 2013). *Actin2* was used as the internal reference. The qRT-PCR was performed using ChamQ Universal SYBR qPCR Master Mix (Vazyme, Nanjing, China) on a Bio-Rad CFX96 Touch real-time detection system (Bio-Rad, United States). Each experiment was performed in triplicates. The expression levels of candidate genes were analyzed using the 2^−△△CT^ method (Livak and Schmittgen, 2001).

## Results

### Phenotypic evaluation of the parents

To accurately evaluate differences in leaf morphology among the three parents, a representative leaf from each node was selected and used to calculate the parameters of leaf length to width ratio (LS). As expected, leaf morphology contrasted between JD12 and SN14, as indicated by their respective LS indices ranging from 1.28 to 1.58 and from 2.90 to 3.49, respectively. These results agree with the categorization that JD12 and SN14 have ovate and lanceolate leaves, respectively. Interestingly, the LS indices of JD17 ranged from 1.40 to 3.80, with the bottom 3 ~ 4 leaves being ovate leaves (LS: 1.48 ~ 1.55), and most of the top 3 ~ 4 leaves being lanceolate leaves (LS: 2.63 ~ 3.44). These results showed considerable heterophylly in JD17 (Figure 1B).

Values derived from measurements of at least ten representative plants revealed RLS indices of 1.96 for JD17, 0.99 for JD12, and 1.14 for SN14 (Figure 1C). These results further confirmed the strong heterophylly of JD17.

### Phenotypic variation among RILs

**Figure 2:**
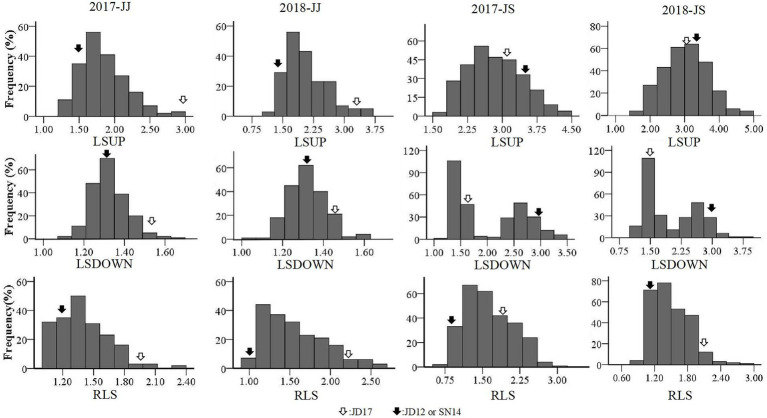
Frequency distributions of leaf-related characteristics obtained from the two RIL populations in the two cropping seasons.

Phenotypic variations of LSUP, LSDOWN, and RLS from the two RIL populations are summarized in Table 1. Coefficients of variation (CV) values for these traits ranged from 0.07 to 0.32. Transgressive segregation was apparent for each of the traits in both populations assessed. The mean values for either of the RIL populations fell between the average parent values, and the maximum and minimum values fell beyond the extremes of the parent values. Broad-sense heritability (*H^2^*) for RLS estimated from the JJ and JS populations was 0.62 and 0.92, respectively. For LSUP, the *H^2^* estimated from the two populations was 0.70 and 0.91, respectively; and the *H^2^* for LSDOWN estimated from the two populations was 0.75 and 0.99, respectively. These results indicated that the phenotypic variation observed in both populations was mainly caused by genetic variation, especially in the JS population (Table 1). Based on values of Kurtosis and Skewness, all tested traits, except for LSDOWN in JS, fit into normal distributions, indicating that they were quantitative traits (Table 1, Figure 2).

**Table 1:** Phenotypic variation in leaf morphology in two RIL populations.

Population	Traits^a^	Parents		RIL							
		JD17	JD12/SN14	Year	Mean ± SD (%)	Min	Max	CV/%^b^	Skew	Kurt	*H*^2^%^c^
JD17 × JD12 (JJ)	RLS	2.0	1.2	2017	1.40 ± 0.25	1.0	2.3	18	0.9	0.9	62.40
		2.2	1.0	2018	1.57 ± 0.37	0.9	2.7	24	0.7	−0.1	
	LSUP	3.0	1.5	2017	1.85 ± 0.35	1.2	3.4	19	1.1	2.0	69.95
		3.1	1.3	2018	2.08 ± 0.53	1.2	3.9	25	1.0	0.7	
	LSDOWN	1.5	1.3	2017	1.33 ± 0.09	1.1	1.6	7	0.6	1.2	74.84
		1.4	1.3	2018	1.32 ± 0.10	1.0	1.7	8	0.5	1.2	
JD17 × SN14 (JS)	RLS	2.0	1.2	2017	1.49 ± 0.34	0.8	2.9	23	0.8	0.7	92.37
		2.2	1.1	2018	1.62 ± 0.46	0.7	3.1	28	0.5	−0.5	
	LSUP	3.0	3.3	2017	2.84 ± 0.61	1.6	4.4	21	0.3	−0.5	90.92
		3.1	3.3	2018	3.06 ± 0.64	1.8	4.8	21	0.3	−0.3	
	LSDOWN	1.5	2.9	2017	2.01 ± 0.64	1.2	3.5	32	0.4	−1.5	98.62
		1.4	3.0	2018	2.02 ± 0.63	1.2	3.8	31	0.5	−1.1	

a RLS, leaf shape ratio; LSUP, top pinnate leaf shape; LSDOWN; basal ternate pinnate leaf shape.

b CV, coefficient of variation.

c H^2^, broad-sense heritability.

### Construction of high-density linkage maps

Based on the GBS sequences obtained, 14,858 high-quality SNPs were detected for the JJ population, which contained 2,491 SNPs that were significantly distorted (*p* < 0.05). These markers formed 2,041 bins. With the use of one representative marker from each of these bins, a linkage map covering a total length of 2,592.6 cM was obtained for this population. The numbers of makers on each of the linkage groups varied from 133 (chromosome 18) to 63 (chromosome 11) with an average of 102.1. Linkage distances covered by these markers varied between 229.9 cM (chromosome 06) and 80.9 cM (chromosome 01) with an average of 129.6 cM. The average distance between adjacent markers was 1.3 cM.

The GBS sequence data produced 12,845 high-quality SNPs in the JS population (including 800 significantly distorted ones), and they formed 2,109 bins. With the use of one representative marker from each of these bins, a linkage map covering a total length of 2,564.4 cM was obtained for JS. The numbers of makers on each of the linkage groups varied from 55 (chromosome 12) to 175 (chromosome 13) with an average of 105.5. Linkage distances covered by these markers varied from 95.7 cM (chromosome 14) and 175.8 cM (chromosome 13) with an average of 128.2 cM. The average distance between adjacent markers was 1.3 cM (Supplementary Table S1).

### Identification of QTL for heterophylly and leaf shape

**Figure 3:**
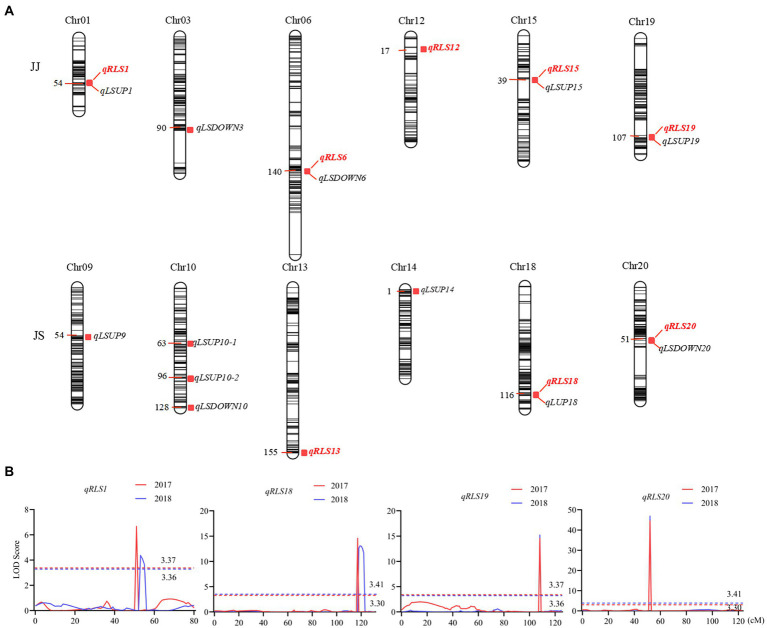
QTL controlling leaf shape characteristics in the two RIL populations. **(A)** QTL hotspots of leaf characteristics in the two RIL populations. The color intensity of the bar chart represents marker density. The number on the left indicates genetic distances in centimorgan (cM). QTL names are shown on the right. **(B)** LOD curves of the four QTL for the ratio of the leaf shape index (RLS). Different colors represent different environments. The curve for data from the 2017 cropping season is in red and that from 2018 in blue. The dashed line indicates the threshold LOD score. The x-axis shows genetic positions in the chromosome.

Eight QTL for heterophylly (RLS) were detected from the two populations over two consecutive seasons (Table 2; Figure 3). Five of them were identified in the JJ population, and they were located on chromosomes 1, 6, 12, 15, and 19, respectively. Phenotypic variances explained by these loci ranged from 5.0 to 27.0% and favorable alleles for all these loci were derived from JD17 except that for *qRLS6*. *qRLS1*, *qRLS6*, *qRLS15,* and *qRLS19* were identified in 2017 and they explained a total of 43.2% phenotypic variance. *qRLS1*, *qRLS12,* and *qRLS19* were identified in 2018 and they explained a total of 40.4% phenotypic variance. Both *qRLS1* and *qRLS19* were detected in both cropping seasons, and *qRLS19* with a maximum LOD value of 15.2 and explained up to 27.0% of phenotypic variance. Three QTL (*qRLS13*, *qRLS18,* and *qRLS20)* for RLS were detected in the JS population. Phenotypic variances explained by them varied from 2.7 to 47.2%. Favorable alleles for those loci were all derived from JD17 except that for *qRLS13*. Both *qRLS18* and *qRLS20* were identified in two consecutive seasons. *qRLS20* explained the highest phenotypic variance (*R^2^* = 47.2%).

**Table 2:** QTL identified for three leaf shape related traits under two-environments based on bin markers genetic map.

Chr	QTL	Traits^a^	Pop.	Years	Marker interval	LOD^b^	PVE^c^ (%)	Add^d^	Novel^e^	Reported QTLs
1	q*RLS1*	RLS	JJ	2017/2018	Gm01:49026799-Gm01:49131130/Gm01:49432006-Gm01:49761132	6.66/4.21	9.08/6.50	0.08/0.08	Yes	
	*qLSUP1*	LSUP	JJ	2017	Gm01:49432006-Gm01:49761132	8.17	13.22	0.13	Yes	
3	*qLSDOWN3*	LSDOWN	JJ	2017/2018	Gm03:38132250-Gm03:38684678/Gm03:38892480-Gm03:39013184	3.60/3.99	8.19/9.47	0.02/0.03	No	Wang et al. (2019)
6	*qRLS6*	RLS	JJ	2017	Gm06:19380218-Gm06:19486680	5.04	6.92	−0.07	Yes	
	*qLSDOWN6*	LSDOWN	JJ	2017	Gm06:19788772-Gm06:34621845	4.55	11.28	0.03	Yes	
9	*qLSUP9*	LSUP	JS	2018	Gm09:6518464-Gm09:6766079	3.53	2.53	0.11	Yes	
10	*qLSUP10-1*	LSUP	JS	2018	Gm10:39068771-Gm10:39093320	4.42	3.19	−0.12	Yes	
	*qLSUP10-2*	LSUP	JS	2017/2018	Gm10:45004437-Gm10:45159991	6.58/6.98	4.55/5.17	0.13/0.16	No	Kim et al. (2005)
	*qLSDOWN10*	LSDOWN	JS	2018	Gm10:50330569-Gm10:50355968	3.79	1.5	0.08	Yes	
12	*qRLS12*	RLS	JJ	2018	Gm12:3549384-Gm12:3738868	4.21	6.5	0.08	Yes	
13	*qRLS13*	RLS	JS	2017	Gm13:42147756-Gm13:42659163	3.37	2.71	−0.05	Yes	
14	*qLSUP14*	LSUP	JS	2018	Gm14:32009-Gm14:277700	5.26	3.84	0.14	Yes	
15	*qRLS15*	RLS	JJ	2017	Gm15:8280651-Gm15:9085621	3.63	5.01	0.06	Yes	
	*qLSUP15*	LSUP	JJ	2017	Gm15:8280651-Gm15:9085621	3.61	5.4	0.08	Yes	
18	*qRLS18*	RLS	JS	2017/2018	Gm18:55570282-Gm18:55724732/Gm18:55804271-Gm18:56171564	14.57/13.08	11.74/10.22	0.11/0.15	Yes	
	*qLSUP18*	LSUP	JS	2017/2018	Gm18:55570282-Gm18:55724732/Gm18:55804271-Gm18:56171564	12.86/25.02	9.61/22.44	0.20/0.33	No	Kim et al. (2005)
19	*qRLS19*	RLS	JJ	2017/2018	Gm19:45157936-Gm19:45286223	14.53/15.20	22.17/27.02	0.12/0.17	Yes	
	*qLSUP19*	LSUP	JJ	2017/2018	Gm19:45157936-Gm19:45286223	10.55/16.07	17.08/28.87	0.14/0.29	No	Kim et al. (2005)
20	*qRLS20*	RLS	JS	2017/2018	Gm20:35767199-Gm20:35910712	44.49/46.85	46.39/47.16	0.22/0.33	No	Sawada (1988)
	*qLSDOWN20*	LSDOWN	JS	2017/2018	Gm20:35767199-Gm20:35910712	99.14/86.99	76.78/72.72	−0.61/−0.56		

a RLS, leaf shape ratio; LSUP, top pinnate leaf shape; LSDOWN, basal ternate pinnate leaf shape.

b LOD, logarithm of odds.

c PVE, phenotypic variation estimated from marker regression against phenotype.

d Add, additive effect of QTL. + and−: Positive values indicate that the JD17 allele increased the trait value and negative values indicate that the JD12 or SN14 allele increased the trait value.

e Novel QTL were determined based on Soybase (http://Soybase.org).

The numbers of loci detected for LSUP and LSDOWN were 8 and 4, respectively. In the JS population, *qRLS20* was located in the same chromosomal interval with *qLSDOWN20,* and *qRLS18* was located in the same chromosomal interval with *qLSUP18* which controls apical leaf shape. In the JJ population, *qRLS1* and *qRLS19* were located in similar intervals with *qLSUP1* and *qLSUP19*, respectively.

Results from the interaction analysis showed that the contributions of Q × E were comparable to those of additive contribution for *qRLS01* and *qRLS18*. The additive contributions of *qRLS19* and *qRLS20* were higher than those of interactive effects between additive effects and environments (Supplementary Table S8).

### Whole genome resequencing of the parents

Three parental lines were resequenced at the whole-genome scale. The numbers of high-quality reads obtained from JD17, JD12, and SN14 were 144.3, 140.1, and 147 million, respectively. About 97.7% of reads were mapped to the reference genome Wm82.a2.v1^6^ for JD17, 97.7% for JD12, and 97.9% for SN14. The average depths of JD17, JD12, and SN14 were 16.7×, 16.1×, and 17.3×, respectively.

A total of 1,445,750 high-quality single-nucleotide variants (SNVs; except intergenic regions) were identified between JD17 and JD12. Of these, 36,952 were non-synonymous mutations, 1,000 were stop-gain, and 246 were stop-loss mutations. Of the 224,799 InDels identified, 2,725 were in coding sequences. 706,626 high-quality SNVs (except intergenic regions) were identified between JD17 and SN14. Of these, 58,792, 813, and 199 were non-synonymous, stop-gain and stop-loss mutations, respectively. Of the 288,824 InDels identified, 3,004 of which were in coding sequences.

### Candidate genes for stable QTL controlling heterophylly

Based on the Williams 82 soybean reference genome (Glyma.Wm82. a2. v1), 23, 49, 17, and 7 annotated genes were discovered in the four major QTL intervals located on chromosomes 1, 18, 19, and 20, respectively (Supplementary Table S2). Among the 96 annotated genes, 81 were found to have at least one GO annotation, which were predicted to be related to various biological processes (Supplementary Table S3).

**Figure 4:**
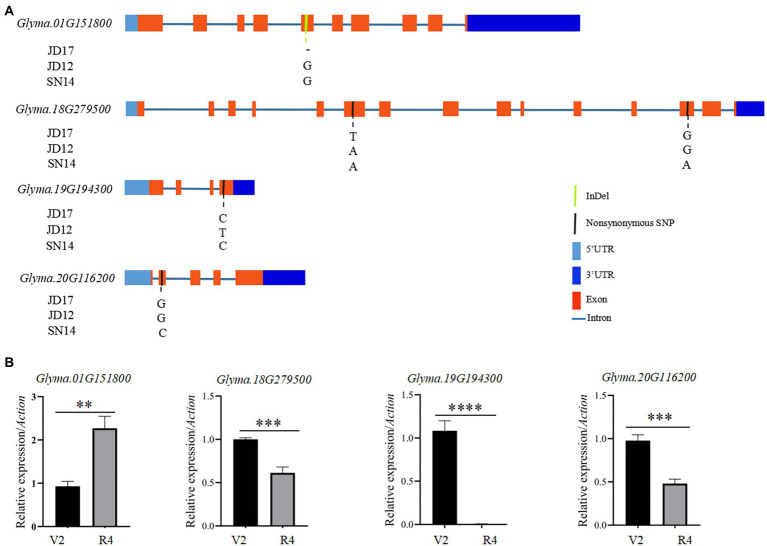
Sequence differences of the four candidate genes among the three parental genotypes and their transcript levels at different developmental stages of JD17. **(A)** Gene structures and genome sequence alignments (non-synonymous SNPs) of the four candidate genes among the three parental genotypes; and **(B)** Expression of the four candidate genes at vegetative growth stages V2 and R4 in JD17. **indicates significant difference at *p* < 0.01, ***significant at *p* < 0.001, and ****significant at *p* < 0.0001.

To identify candidate genes underlying QTL for heterophylly, we re-sequenced the three parents. Differences in sequences within the four targeted QTL intervals between the parents for both populations were identified. A total of 205 SNPs were identified and 99 of them were non-synonymous mutations. Six InDels were detected in the exons regions (Supplementary Tables S4, S5). Within the qRLS01 interval, 44 SNPs in the exon sequences of 11 genes were identified. Of these, 36 were non-synonymous mutations, and one of them was a stop-gain mutation in *Glyma.01G153900* (Supplementary Tables S4, S6). One InDel located in Glyma.01G151800 caused a frameshift mutation (Supplementary Table S5). Within the qRLS18 interval, 103 SNPs in the exon sequences of 30 genes were identified. Of these, 61 were non-synonymous mutations (Supplementary Table S4). The SNPs in *Glyma.18G277000*, *Glyma.18G277400,* and *Glyma.18G279000* were stop-gain mutations (Supplementary Table S6). Within the qRLS19 interval, 25 SNPs in the exon sequences of 11 genes were detected. Of these, 12 were non-synonymous mutations and they were located in nine genes (Supplementary Table S4). Five InDels were identified in three genes (Supplementary Table S5). Within the qRLS20 interval, three SNPs occurred in the exon sequences of three genes. One of them was a non-synonymous mutation (G → C transversion) in exons of *Glyma.20G116200*, producing an amino acid substitution (Aspartic acid→Histidine; Figure 4A; Table 3).

**Table 3:** Candidate genes for heterophylly in soybean.

QTL name	Candidate gene	GO biological process descriptions	GO molecular function descriptions	Homologous genes in *Arabidopsis*
q*RLS1*	*Glyma.01G151800*	Regulation of shoot system development	Extracellular region; nucleus; cytoplasm	AT5G45300, *BETA-AMYLASE 8* (*BAM*8)
q*RLS18*	*Glyma.18G279500*	Cell differentiation; cell division	Protein binding; ubiquitin protein ligase activity;	AT2G20000, *HOBBIT* (*CDC27*)
q*RLS19*	*Glyma.19G194300*	Vegetative to reproductive phase transition of meristem; cell differentiation; meristem determinacy	Transcription coregulator activity;	AT5G03840, *TERMINAL FLOWER 1* (*TFL1*)
q*RLS20*	*Glyma.20G116200*	Leaf morphogenesis; specification of floral organ identity; abaxial cell fate specification; carpel development; stamen development; anther development; flower development;	Nucleic acid binding; DNA-binding transcription factor activity; protein binding; metal ion binding;	AT1G68480, *JAGGED* (*JAG*)

To further determine whether candidate genes play a role in heterophytosis, we compared the expression of *Glyma.01G151800*, *Glyma.18G279500*, *Glyma.18G279600*, *Glyma.19G194100*, *Glyma.19G194300*, and *Glyma.20G116200* in the shoot of JD17 at different developmental stages. The results showed that the expression of *Glyma.18G279500*, *Glyma.19G194300,* and *Glyma.20G116200* was significantly downregulated in terminal buds at R4 compared with those at V2 stage. *Glyma.01G151800* was significantly upregulated at R4 compared with that at V2 stage, and the expressions of *Glyma.18G279600* and *Glyma.19G194100* were not significantly different between these two developmental stages (Figure 4B). Combined with the data from gene annotation analysis, we believe that *Glyma.01G151800*, *Glyma.18G279500*, *Glyma.19G194300*, and *Glyma.20G116200* were candidate genes for heterophylly in soybean.

## Discussion

Plant architecture is a critical and complex trait that could significantly influence crop yield. Leaves are the main organs for photosynthesis and synthesize chemical compounds needed throughout crop development. As a major component of plant architecture, it is not surprising that leaf morphology and their arrangements in the canopy affect the efficiencies of light capture and photosynthesis (Chitwood and Sinha, 2016). As such, they have attracted increasing attention from breeders. Heterophylly refers to two or more types of leaves on the same plant. It may be the result of a rigid development program or a plastic response of plants to different habitat conditions (Kerstetter and Poethiq, 1998; Titus et al., 2001; Hejnák et al., 2014). Few studies on heterophylly have been reported in soybean, and the genetic and molecular mechanisms remain unknown. In order to investigate the genetic characteristics of heterophylly, we constructed two RIL populations, one by crossing JD17 (heterophylly) with JD12 (ovate leaves) and the other between JD17 and SN14 (lanceolate leaves). Assessments of phenotypes for two consecutive cropping seasons revealed that the heritability of heterophylly in these two populations was 62.40 and 92.37%, respectively, (Table 2), and the segregations of the RLS fit normal or skew-normal distribution models (Figure 2).

To understand the genetics of heterophylly, we identified QTL associated with leaf shape in this study. We detected 18 QTL for LSUP, LSDOWN, and RLS. We compared the QTL detected in this study with those reported previously. Based on their physical positions in the soybean reference genome, 6 leaf shape-related QTL identified in this study co-localized with QTL or genes reported in previous studies. For example, *qLSDOWN20*, the major QTL for basal leaf shape in the JS population, had the same position with *Ln* (Sawada, 1988; Fang et al., 2013). QTL controlling the shapes of the top three leaves, *qLSUP10-2*, *qLSUP18*, and *qLSUP19,* were in the same genetic region as the QTL reported by Kim et al. (2005). *qLSDOWN3* was at a similar position as the *qLS3h* reported by Wang et al. (2019). *qLSUP1, qLSUP9, qLSUP10-1,qLSUP14*, *qLSUP15, qLSDOWN6,* and *qLSDOWN10* were novel QTL (Table 2). As this study is the first to examine QTL related to heterophylly, all eight QTL (five detected in JJ population and three in JS population) were identified for the first time relative to heterophylly. Of these QTL, the intervals of *qRLS12*, *qRLS18*, *qRLS19,* and *qRLS20* overlap with reported QTL for leaf shape. The intervals of *qRLS1* and *qRLS15* have not been reported to be associated with leaf shape in any previous studies.

Due likely to gametophytic competition or sporophytic selection (O’Donoughue et al., 1992), segregation distortions of molecular markers are a phenomenon widely reported in various plant species (Liu et al., 1996; Konduri et al., 2000). With the concern that they may cause issues, distorted loci were sometimes discarded in QTL detection (Su et al., 2017). However, the effects of distorted loci on QTL detection may not be as negative as these authors assumed. In fact, many studies found that they have little effects on QTL detection (Hackett and Broadfoot, 2003; Ma et al., 2022). Some studies even found that their inclusion may benefit QTL detection (Xu, 2008; Zhang et al., 2010; Zuo et al., 2019). Deleting such loci may cause potential QTL to be missed, or a change in QTL location (Shah et al., 2014). We thus included all distorted loci in linkage map construction and in QTL detection in the study reported here.

The difference in leaf shape between JD17 and JD12 is mainly in the apical leaves. The difference between JD17 and SN14 (which has lance-leaved shape) is mainly in the basal leaves. By comparing the QTL for LSUP, LSDOWN, and RLS between the two populations, it was found that the QTL on chromosomes 1 and 19 affected both apical leaf shape and heterophylly in the JJ population. In the JS population, the QTL on chromosome 20 affected both basal leaf shape and heterophylly, and the QTL on chromosome 18 affected both apical leaf shape and heterophylly. Therefore, we have concluded that *qRLS20* regulates basal leaf shape at an early stage (nutritional growth), that *qRLS1*, *qRLS18,* and *qRLS19* regulate apical leaf shape, and that JD17 possesses superior allelic variation at these QTL loci in producing heterophylly phenotype.

Based on gene annotations, sequence analysis of parental genes, and qRT-PCR, we identified six candidate genes for heterophylly in the intervals of the stable QTL. One of them, *Glyma.01G151800* was homologous to *AtBMY8* (*BETA-AMYLASE* 8) that was related to leaf growth and development in *Arabidopsis thaliana*. Compared with the wild type, *AtBAM8-*OE plants have short petioles and rounded, dark-green, and hyponastic leaves (Reinhold et al., 2011). *Glyma.20G116200* (*Ln*), which encodes *GmJAGGED1* (*GmJAG1*), is a major gene regulating leaf shape in soybean. A single base substitution of the *Ln* gene from guanine (G) to cytosine (C) led to an amino acid change in the conserved EAR motif of *GmJAG1*, resulting in the loss of function of the gene (Jeong et al., 2012; Fang et al., 2013) and the leaves change from round to lanceolate (Cai et al., 2021). The comparison of parental gene sequences showed that JD17 contained *Ln* allelic variation and SN14 contained *ln* allelic variation. In *Arabidopsis,* an increase in the length/width ratio of the leaf blade is a marker for vegetative phase change (Telfer et al., 1997). The change in leaf morphology is a marker for phase transition from juvenile to adult, due mainly to the temporal development of the shoot apical meristem (Silva et al., 2019). *Glyma.19G194300* (*Dt1*) is a homolog of the *TERMINAL FLOWER1* (*TFL1*) gene in *Arabidopsis* which plays diverse roles related to signaling pathways controlling growth and differentiation (Thompson et al., 1995; Liu et al., 2010). In *Arabidopsis*, *TFL1* participates in the transition from vegetative to reproductive phases. *Glyma.18G279500* was also involved in cell differentiation and cell division.

In summary, this report elucidated the genetic characteristics and identified QTL and candidate genes underlying the characteristics of heterophylly. Results obtained in this study provides preliminary information on potential roles for genetic mechanism associated with heterophylly. These results lay the foundation for further efforts to identify valuable genetic resources for MAS breeding programs.

## Data availability statement

The data presented in the study are deposited in the NCBI BioProject repository, accession number PRJNA848633.

## Author contributions

QC, CY, and MZ designed the experiments and analyzed the data. QC, LA, BL, YW, YF, and JL conducted the experiments. LY, QC, and XS constructed and genotyped the mapping populations. QC, HZ, and CL were mainly responsible in drafting the manuscript with contributions from all other authors. All authors contributed to the article and approved the submitted version.

## Funding

This work reported in this publication is supported by the China Agriculture Research System of MOF and MARA (CARS-04-PS06 for CY), the National Natural Science Foundation of China (31471522 for MZ), the Science and Technology Innovation Team of Soybean Modern Seed Industry in Hebei Province (21326313D-4 for LY), and the Hebei Natural Science Foundation (C2020301020 for MZ).

## Conflict of interest

The authors declare that the research was conducted in the absence of any commercial or financial relationships that could be construed as a potential conflict of interest.

## Publisher’s note

All claims expressed in this article are solely those of the authors and do not necessarily represent those of their affiliated organizations, or those of the publisher, the editors and the reviewers. Any product that may be evaluated in this article, or claim that may be made by its manufacturer, is not guaranteed or endorsed by the publisher.

## Footnotes

^1^ https://www.soybase.org/sitemap.php

^2^ https://phytozome-next.jgi.doe.gov/info/Gmax_Wm82_a2_v1

^3^ https://www.isbreeding.net/software/

^4^ http://www.soybase.org/

^5^ http://picard.sourceforge.net

^6^ http://www.soybase.org/

## References

[ref1] Baldocchi D. D. Hutchison B. A. Matt D. R. Mcmillen R. T. (1985). Canopy radiative transfer models for spherical and known leaf inclination angle distributions: a test in an Oak-Hickory forest. J. Appl. Ecol. 22, 539–555. doi: 10.2307/2403184

[ref2] Beydler B. D. Osadchuk K. Cheng C. L. Manak J. R. Irish E. E. (2016). The juvenile phase of maize sees upregulation of stress-response genes and is extended by exogenous Jasmonic Acid. Plant Physiol. 171, 2648–2658. doi: 10.1104/pp.15.01707, PMID: 27307257 PMC4972259

[ref3] Beyschlag J. Zotz G. (2017). Heteroblasty in epiphytic bromeliads: functional implications for species in understorey and exposed growing sites. Ann. Bot. 120, 681–692. doi: 10.1093/aob/mcx048, PMID: 28510657 PMC5691803

[ref4] Bhagsari A. S. Brown R. H. (1986). Leaf photosynthesis and its correlation with leaf area. Crop Sci. 26, 127–132. doi: 10.2135/cropsci1986.0011183X002600010030x

[ref5] Cai Z. D. Xian P. Q. Cheng Y. B. Ma Q. B. Lian T. X. Nian H.. (2021). CRISPR/Cas9-mediated gene editing of GmJAGGED1 increased yield in the low latitude soybean variety Huachun 6. Plant Biotechnol. J. 19, 1898–1900. doi: 10.1111/pbi.13673, PMID: 34289223 PMC8486244

[ref6] Cheng W. Liu F. Li M. Hu X. Chen H. Pappoe F.. (2015). Variation detection based on next-generation sequencing of type Chinese 1 strains of *Toxoplasma gondii* with different virulence from China. BMC Genomics 16, 888. doi: 10.1186/s12864-015-2106-z, PMID: 26518334 PMC4628340

[ref7] Chitwood D. H. Ranjan A. Kumar R. Ichihashi Y. Zumstein K. Headland L. R.. (2014). Resolving distinct genetic regulators of tomato leaf shape within a heteroblastic and ontogenetic context. Plant Cell 26, 3616–3629. doi: 10.1105/tpc.114.130112, PMID: 25271240 PMC4213164

[ref8] Chitwood D. H. Sinha N. R. (2016). Evolutionary and environmental forces sculpting leaf development. Curr. Biol. 26, R297–R306. doi: 10.1016/j.cub.2016.02.033, PMID: 27046820

[ref9] Dinkins R. D. Keim K. R. Farno L. Edwards L. H. (2002). Expression of the narrow leaflet gene for yield and agronomic traits in soybean. J. Hered. 93, 346–351. doi: 10.1093/jhered/93.5.346, PMID: 12547923

[ref10] Eshed Y. Zamir D. (1995). An introgression line population of *Lycopersicon pennellii* in the cultivated tomato enables the identification and fine mapping of yield-associated QTL. Genetics 141, 1147–1162. doi: 10.1093/genetics/141.3.1147, PMID: 8582620 PMC1206837

[ref11] Fang C. Li W. Y. Li G. Q. Wang Z. Zhou Z. K. Ma Y. M.. (2013). Cloning of Ln gene through combined approach of map-based cloning and association study in soybean. J. Genet. Genom. 40, 93–96. doi: 10.1016/j.jgg.2013.01.002, PMID: 23439408

[ref12] Givnish T. J. (1988). Adaptation to sun and shade: a whole-plant perspective. Aust. J. Plant Physiol. 15, 63–92. doi: 10.1071/pp9880063

[ref13] Graham P. H. Vance C. P. (2003). Legumes: importance and constraints to greater use. Plant Physiol. 131, 872–877. doi: 10.1104/pp.017004, PMID: 12644639 PMC1540286

[ref14] Hackett C. A. Broadfoot L. B. (2003). Effects of genotyping errors, missing values and segregation distortion in molecular marker data on the construction of linkage maps. Heredity 90, 33–38. doi: 10.1038/sj.hdy.6800173, PMID: 12522423

[ref15] Hejnák V. Hniličková H. Hnilička F. (2014). Effect of ontogeny, heterophylly and leaf position on the gas exchange of the hop plant. Plant Soil Environ. 60, 525–530. doi: 10.17221/671/2014-PSE

[ref16] Jeong N. Suh S. J. Kim M. H. Lee S. Moon J. K. Kim H. S.. (2012). Ln is a key regulator of leaflet shape and number of seeds per pod in soybean. Plant Cell 24, 4807–4818. doi: 10.1105/tpc.112.104968, PMID: 23243125 PMC3556959

[ref17] Jun T. H. Freewalt K. Michel A. P. Mian R. Singh R. (2014). Identification of novel QTL for leaf traits in soybean. Plant Breed. 133, 61–66. doi: 10.1111/pbr.12107

[ref18] Kerstetter R. A. Poethiq R. S. (1998). The specification of leaf identity during shoot development. Annu. Rev. Cell Dev. Biol. 14, 373–398. doi: 10.1146/annurev.cellbio.14.1.373, PMID: 9891788

[ref19] Kim H. K. Kang S. T. Suh D. Y. (2005). Analysis of quantitative trait loci associated with leaflet types in two recombinant inbred lines of soybean. Plant Breed. 124, 582–589. doi: 10.1111/j.1439-0523.2005.01152.x

[ref20] Konduri V. Godwin I. Liu C. J. (2000). Genetic mapping of the *Lablab purpureus* genome suggests the presence of ‘cuckoo’ gene(s) in this species. Theor. Appl. Genet. 100, 866–871. doi: 10.1007/s001220051363

[ref21] Li H. Durbin R. (2009). Fast and accurate short read alignment with Burrows-Wheeler transform. Bioinformatics 25, 1754–1760. doi: 10.1093/bioinformatics/btp324, PMID: 19451168 PMC2705234

[ref22] Li H. Handsaker B. Wysoker A. Fennell T. Ruan J. Homer N.. (2009). The sequence alignment/map format and SAMtools. Bioinformatics 25, 2078–2079. doi: 10.1093/bioinformatics/btp352, PMID: 19505943 PMC2723002

[ref23] Li H. H. Ribaut J. M. Li Z. L. Wang J. K. (2008). Inclusive composite interval mapping (ICIM) for digenic epistasis of quantitative traits in biparental populations. Theor. Appl. Genet. 116, 243–260. doi: 10.1007/s00122-007-0663-5, PMID: 17985112

[ref24] Liu D. L. Chen S. W. Liu X. C. Yang F. Liu W. G. She Y. H.. (2019). Genetic map construction and QTL analysis of leaf-related traits in soybean under monoculture and relay intercropping. Sci. Rep. 9, 2716. doi: 10.1038/s41598-019-39110-8, PMID: 30804368 PMC6390081

[ref25] Liu C. J. Devos K. M. Witcombe J. R. Pittaway T. S. Nash M. Gale M. D. (1996). The effect of genome and sex on recombination in *Pennisetum* species. Theor. Appl. Genet. 93, 902–908. doi: 10.1007/BF00224092, PMID: 24162424

[ref26] Liu Y. B. Li X. R. Chen G. X. Li M. M. Liu M. L. Liu D. (2015). Epidermal micromorphology and mesophyll structure of *Populus euphratica* heteromorphic leaves at different development stages. PLoS One 10:e0141578. doi: 10.1371/journal.pone.0137701, PMID: 26496643 PMC4619683

[ref27] Liu B. H. Watanabe S. Uchiyama T. Kong F. J. Kanazawa A. Xia Z. J.. (2010). The soybean stem growth habit gene *Dt1* is an ortholog of Arabidopsis *TERMINAL FLOWER*^1^. Plant Physiol. 153, 198–210. doi: 10.1104/pp.109.150607, PMID: 20219831 PMC2862436

[ref28] Livak K. J. Schmittgen T. D. (2001). Analysis of relative gene expression data using real-time quantitative PCR and the 2(-Delta Delta C(T)) method. Methods 25, 402–408. doi: 10.1006/meth.2001.1262 11846609

[ref29] Ma C. H. Rehman A. Li H. G. Zhao Z. B. Sun G. F. Du X. M. (2022). Mapping of dwarfing QTL of Ari1327, a semi-dwarf mutant of upland cotton. BMC Plant Biol. 22, 5. doi: 10.1186/s12870-021-03359-x, PMID: 34979924 PMC8722190

[ref30] Meng L. Li H. H. Zhang L. Y. Wang J. K. (2015). QTL IciMapping: integrated software for genetic linkage map construction and quantitative trait locus mapping in biparental populations. Crop J. 3, 269–283. doi: 10.1016/j.cj.2015.01.001

[ref31] O’Donoughue L. S. Wang Z. Röder M. Kneen B. Leggett M. Sorrells M. E.. (1992). An RFLP-based linkage map of oats based on a cross between two diploid taxa (*Avena atlantica* × *A. hirtula*). Genome 35, 765–771. doi: 10.1139/g92-117

[ref32] Pei R. L. Zhang J. Y. Tian L. Zhang S. R. Han F. X. Yan S. R.. (2018). Identification of novel QTL associated with soybean isoflavone content. Crop J. 6, 244–252. doi: 10.1016/j.cj.2017.10.004

[ref33] Reinhardt D. Kuhlemeier C. (2002). Plant architecture. EMBO Rep. 3, 846–851. doi: 10.1093/embo-reports/kvf177, PMID: 12223466 PMC1084230

[ref34] Reinhold H. Soyk S. Šimková K. Hostettler C. Marafino J. Mainiero S.. (2011). β-amylase-like proteins function as transcription factors in arabidopsis, controlling shoot growth and development. Plant Cell 23, 1391–1403. doi: 10.1105/tpc.110.081950, PMID: 21487098 PMC3101533

[ref35] Rowland S. D. Zunstein K. Nakayama H. Cheng Z. Flores A. M. Chitwood D. H.. (2020). Leaf shape is a predictor of fruit quality and cultivar performance in tomato. New Phytol. 226, 851–865. doi: 10.1111/nph.16403, PMID: 31880321 PMC7187315

[ref36] Sarlikioti V. Visser P. H. Buck-Sorlin G. H. Marcelis L. F. (2011). How plant architecture affects light absorption and photosynthesis in tomato: towards an ideotype for plant architecture using a functional-structural plant model. Ann. Bot. 108, 1065–1073. doi: 10.1093/aob/mcr221, PMID: 21865217 PMC3189847

[ref490] Sawada S. (1988). Inheritance of leaflet shape in soybeans. Soybean Genet. Newsl. 15:61e65., PMID: 18957707

[ref37] Shah R. Cavanagh C. R. Huang B. E. (2014). Computationally efficient map construction in the presence of segregation distortion. Theor. Appl. Genet. 127, 2585–2597. doi: 10.1007/s00122-014-2401-0, PMID: 25260690

[ref38] Silva P. O. Batista D. S. Cavalcanti J. H. F. Koehler A. D. Vieira L. M. Fernandes A. M.. (2019). Leaf heteroblasty in *Passiflora edulis* as revealed by metabolic profiling and expression analyses of the microRNAs miR156 and miR172. Ann. Bot. 123, 1191–1203. doi: 10.1093/aob/mcz025, PMID: 30861065 PMC6612941

[ref39] Smith S. E. Kuehl R. O. Ray I. M. Hui R. Soleri D. (1998). Evaluation of simple methods for estimating broad-sense heritability in stands of randomly planted genotypes. Crop Sci. 38, 1125–1129. doi: 10.2135/cropsci1998.0011183X003800050003x

[ref40] Su C. F. Wang W. Gong S. L. Zuo J. H. Li S. J. Xu S. Z. (2017). High density linkage map construction and mapping of yield trait QTLs in maize (*Zea mays*) using the genotyping-by-sequencing (GBS) technology. Front. Plant Sci. 8:706. doi: 10.3389/fpls.2017.00706, PMID: 28533786 PMC5420586

[ref41] Swarts K. Li H. Navarro A. An D. Romay M. Hearne S.. (2014). Novel methods to optimize genotypic imputation for low-coverage, next-generation sequence data in crop plants. Plant Genome 7, 175–177. doi: 10.3835/plantgenome2014.05.0023

[ref42] Tanaka-Oda A. Kenzo T. Kashimura S. Ninomiya I. Wang L. H. Yoshikawa K.. (2010). Physiological and morphological differences in the heterophylly of Sabina vulgaris ant. In the semi-arid environment of Mu Us Desert, Inner Mongolia, China. J. Arid. Environ. 74, 43–48. doi: 10.1016/j.jaridenv.2009.07.013

[ref43] Telfer A. Bollman K. M. Poethig R. S. (1997). Phase change and the regulation of trichome distribution in *Arabidopsis thaliana*. Development 124, 645–654. doi: 10.1242/dev.124.3.645, PMID: 9043079

[ref44] Thompson J. A. Nelson R. L. Schweitzer L. E. (1995). Relationships among specific leaf weight, photosynthetic rate, and seed yield in soybean. Crop J. 35, 1575–1581. doi: 10.2135/cropsci1995.0011183X003500060010x

[ref45] Titus J. E. Gary Sullivan P. (2001). Heterophylly in the yellow waterlily, *Nuphar variegata* (Nymphaeaceae): effects of [CO2], natural sediment type, and water depth. Amer. J. Bot. 88, 1469–1478. doi: 10.2307/3558455, PMID: 21669680

[ref46] Wang L. Cheng Y. B. Ma Q. B. Mu Y. H. Huang Z. F. Xia Q. J.. (2019). QTL fine-mapping of soybean (*Glycine max* L.) leaf type associated traits in two RILs populations. BMC Genomics 20, 260. doi: 10.1186/s12864-019-5610-8, PMID: 30940069 PMC6444683

[ref47] Wang K. Li M. Hakonarson H. (2010). ANNOVAR: functional annotation of genetic variants from high-throughput sequencing data. Nucl. Acid. Res. 38, e164. doi: 10.1093/nar/gkq603, PMID: 20601685 PMC2938201

[ref48] Xia W. X. Xiao Z. A. Cao P. Zhang Y. Du K. B. Wang N. (2018). Construction of a high-density genetic map and its application for leaf shape QTL mapping in poplar. Planta 248, 1173–1185. doi: 10.1007/s00425-018-2958-y, PMID: 30088086

[ref49] Xu S. (2008). Quantitative trait locus mapping can benefit from segregation distortion. Genetics 180, 2201–2208. doi: 10.1534/genetics.108.090688, PMID: 18957707 PMC2600952

[ref50] Yano S. Terashima I. (2001). Separate localization of light signal perception for sun or shade type chloroplast and palisade tissue differentiation in *Chenopodium album*. Plant Cell Physiol. 42, 1303–1310. doi: 10.1093/pcp/pce183, PMID: 11773522

[ref51] Zeng Q. Wu J. Huang S. Yuan F. Liu S. Wang Q.. (2019). SNP-based linkage mapping for validation of adult plant stripe rust resistance QTL in common wheat cultivar Chakwal 86. Crop J. 7, 176–186. doi: 10.1016/j.cj.2018.12.002

[ref52] Zhang L. Wang S. Li H. Deng Q. Zheng A. Li S.. (2010). Efects of missing marker and segregation distortion on QTL mapping in F2 populations. Theor. Appl. Genet. 121, 1071–1082. doi: 10.1007/s00122-010-1372-z, PMID: 20535442

[ref53] Zhou Z. Q. Zhang C. S. Zhou Y. Hao Z. F. Wang Z. H. Zeng X.. (2016). Genetic dissection of maize plant architecture with an ultra-high density bin map based on recombinant inbred lines. BMC Genomics 17, 178. doi: 10.1186/s12864-016-2555-z, PMID: 26940065 PMC4778306

[ref54] Zuo J. F. Niu Y. Cheng P. Feng J. Y. Han S. F. Zhang Y. H.. (2019). Effect of marker segregation distortion on high density linkage map construction and QTL mapping in soybean (*Glycine max* L.). Heredity 123, 579–592. doi: 10.1038/s41437-019-0238-7, PMID: 31152165 PMC6972858

